# Comparison of NGS and MFC Methods: Key Metrics in Multiple Myeloma MRD Assessment

**DOI:** 10.3390/cancers12082322

**Published:** 2020-08-18

**Authors:** Katharina Kriegsmann, Michael Hundemer, Nicole Hofmeister-Mielke, Philipp Reichert, Calin-Petru Manta, Mohamed H.S. Awwad, Sandra Sauer, Uta Bertsch, Britta Besemer, Roland Fenk, Mathias Hänel, Markus Munder, Katja C. Weisel, Igor W. Blau, Andreas Neubauer, Carsten Müller-Tidow, Marc S. Raab, Hartmut Goldschmidt, Stefanie Huhn, for the German-speaking Myeloma Multicenter Group (GMMG)

**Affiliations:** 1 Department of Hematology, Oncology and Rheumatology, University Hospital Heidelberg, 69120 Heidelberg, Germany; Nicole.Hofmeister-Mielke@med.uni-heidelberg.de (N.H.-M.); Philipp.Reichert@med.uni-heidelberg.de (P.R.); Calin-Petru.Manta@med.uni-heidelberg.de (C.-P.M.); MohamedHemaidSayed.Awwad@med.uni-heidelberg.de (M.H.S.A.); Sandra.Sauer@med.uni-heidelberg.de (S.S.); Uta.Bertsch@med.uni-heidelberg.de (U.B.); Carsten.Mueller-Tidow@med.uni-heidelberg.de (C.M.-T.); Marc.Raab@med.uni-heidelberg.de (M.S.R.); Hartmut.Goldschmidt@med.uni-heidelberg.de (H.G.); Stefanie.Huhn@med.uni-heidelberg.de (S.H.); 2 National Center for Tumor Diseases Heidelberg, 69120 Heidelberg, Germany; 3 Department of Hematology, Oncology and Immunology, University Hospital Tübingen, 72076 Tübingen, Germany; britta.besemer@med.uni-tuebingen.de; 4 Department of Hematology, Oncology and Clinical Immunology, University Hospital Düsseldorf, 40225 Düsseldorf, Germany; Fenk@med.uni-duesseldorf.de; 5 Department of Internal Medicine III, Klinikum Chemnitz, 09113 Chemnitz, Germany; m.haenel@skc.de; 6 Department of Internal Medicine III, University Medical Center Mainz, 55131 Mainz, Germany; munder@uni-mainz.de; 7 Department of Oncology, Hematology and Bone Marrow Transplantation with Department of Pneumology, University Medical Center Hamburg-Eppendorf, 20246 Hamburg, Germany; k.weisel@uke.de; 8 Medical Clinic, Charité University Medicine Berlin, 10117 Berlin, Germany; igor.blau@charite.de; 9 Department of Hematology, Oncology and Immunology, Philipps-University Marburg, 35043 Marburg, Germany; neubauer@med.uni-marburg.de

**Keywords:** multiple myeloma (MM), minimal residual disease (MRD), next-generation sequencing (NGS), multicolor flow cytometry (MFC), EuroFlow

## Abstract

In order to meet the challenges in data evaluation and comparability between studies in multiple myeloma (MM) minimal residual disease (MRD) assessment, the goal of the current study was to provide a step-by-step evaluation of next-generation sequencing (NGS) and multicolor flow cytometry (MFC) data. Bone marrow (BM) sample pairs from 125 MM patients were analyzed by NGS and MFC MM MRD methods. Tumor load (TL) and limit of detection (LOD) and quantification (LOQ) were calculated. The best-fit MRD cut-off was chosen as 1 × 10^−5^, resulting in an overall 9.6% (*n* overall = 12 (NGS *n* = 2, MFC *n* = 10)) nonassessable cases. The overall concordance rate between NGS and MFC was 68.0% (*n* = 85); discordant results were found in 22.4% (11.2% (*n* = 14) of cases in each direction. Overall, 55.1% (*n* = 60/109) and 49.5% (*n* = 54/109) of patients with a serological response ≥ very good partial response (VGPR) showed BM MRD negativity by NGS and MFC, respectively. A good correlation in the TL assessed by both techniques was found (correlation coefficient = 0.8, *n* = 40, *p* < 0.001). Overall, our study shows good concordance between MM BM MRD status and TL when comparing NGS and MFC at a threshold of 10^–5^. However, a sufficient number of analyzed events and calculation of MRD key metrics are essential for the comparison of methods and evaluability of data at a specific MRD cut-off.

## 1. Introduction

Minimal residual disease (MRD) is defined as a small number of malignant cells that persist during or after treatment and cannot be detected by serological or cytological methods. State-of-the-art methods for the highly sensitive and standardized detection of bone marrow (BM) MRD in multiple myeloma (MM) include next-generation sequencing (NGS) and multicolor flow cytometry (MFC). NGS, in the form of an ultradeep targeted sequencing assay, targets immunoglobulin heavy- and light-chain DNA sequences using consensus primers [1]. In MFC, achieved through a two-tube, eight-color antibody panel, the identification of malignant cells is based on an aberrant immunophenotype displayed by neoplastic MM cells compared to normal plasma cells [2]. Based on optimal requirements, including sample quality and sample processing, both MM MRD detection methods have been described to reach a sensitivity of up to one tumor cell per 1,000,000 BM cells (10^−6^) [1,3].

In MM, the presence of residual tumor cells in BM is considered the major cause of relapse. Therefore, MRD diagnostics provide one of the strongest prognostic measurements for outcome and information on treatment efficiency [3,4,5,6]. As demonstrated by numerous studies and subsequent meta-analyses, MRD negativity after treatment is associated with significantly better progression-free and overall survival (PFS, OS) in newly diagnosed and relapsed/refractory MM patients [7,8]. These data strongly support the implementation of MRD status as a primary endpoint and a surrogate for outcome in MM clinical trials. Implementing MRD as a regulatory endpoint is anticipated to result in accelerated drug development in MM as it would allow for faster regulatory approval [9]. Additionally, there are emerging data suggesting that MRD assays have the ability to be implemented as disease monitoring tools and response biomarkers in routine clinical practice [10]. Taking this route, the U.S. Food and Drug Administration (FDA) recently authorized the first NGS assay to detect MRD in MM patients from BM [11].

Aside from the demand for more sensitive MRD assays and clinical trials defining the effect of MRD negativity, key metrics of currently used laboratory methods have to be defined, evaluated and reported in a standardized manner [9,12]. In particular, this includes the comparability of the results obtained from different MRD detection methods and in different trials by reporting the limits of detection (LOD), limits of quantification (LOQ) and the definition of applied MRD cut-offs.

## 2. Results

### 2.1. The Amount of Available Sample Is Crucial and Affects the Evaluation and Comparison of Results at Specific MRD Cut-offs

To compare the NGS and MFC assays, sample pairs from patients (*n* = 125) with available results from both MRD detection methods at the same timepoint of treatment were evaluated. To assess the quality of the performed NGS and MFC analyses, key validation metrics were calculated.

**Figure 1:**
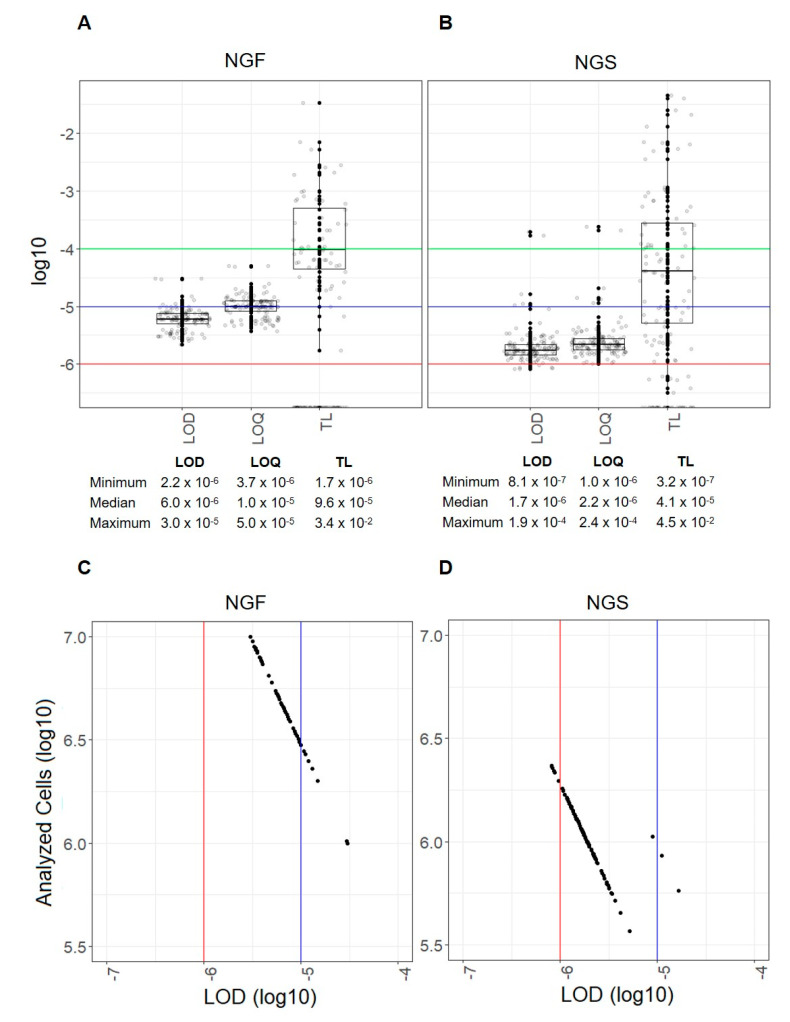
Key metrics of multicolor flow cytometry (MFC) and next-generation sequencing (NGS) analyses. The tumor load (TL) with the corresponding limit of detection (LOD) and limit of quantification (LOQ) is shown for each sample analyzed with (**A**) MFC and (**B**) NGS. The horizontal lines represent different possible minimal residual disease (MRD) cut-offs (1 × 10^−4^ green, 1 × 10^−5^ blue and 1 × 10^−6^ red). Descriptive statistics (minimum, median, maximum) for each key metric are shown below the figure. The number of analyzed cells and the corresponding LODs are shown for each sample on a log10 scale for (**C**) MFC and (**D**) NGS analysis. The vertical lines represent different possible MRD cut-offs (1 × 10^−5^ blue and 1 × 10^−6^ red).

In NGS, the median number of cell equivalents analyzed was 1.1 × 10^6^ (1.8 × 10^5^–2.3 × 10^6^). This resulted in a median NGS LOD level of 1.7 × 10^−6^ (8.1 × 10^−7^–1.9 × 10^−4^) and a median NGS LOQ level of 2.2 × 10^−6^ (1.0 × 10^−6^–2.4 × 10^−4^). Tumor cells were detectable in 74.4% (*n* = 93) of patients. The median detected NGS TL in the overall cohort was 4.1 × 10^−5^ (3.2 × 10^−7^–4.5 × 10^−2^), as shown in Figure 1A. In MFC analyses, a median of 5.0 × 10^6^ (1.0 × 10^6^–1.4 × 10^7^) events was analyzed, and a median MFC LOD of 6.0 × 10^−6^ (2.2 × 10^−6^–3.0 × 10^−5^) and a median MFC LOQ of 1.0 × 10^−5^ (3.7 × 10^−6^–5.0 × 10^−5^) were reached. Tumor cells were detectable in 48.0% (*n* = 60) of patients. Irrespective of MRD-negative cases, the median MFC TL in the overall cohort was 9.6 × 10^−5^ (1.7 × 10^−6^–3.4 × 10^−2^), as shown in Figure 1B. Comparing the number of cells necessary to reach sufficient LOD levels, NGS required fewer cells than MFC (Figure 1C,D).

**Figure 2:**
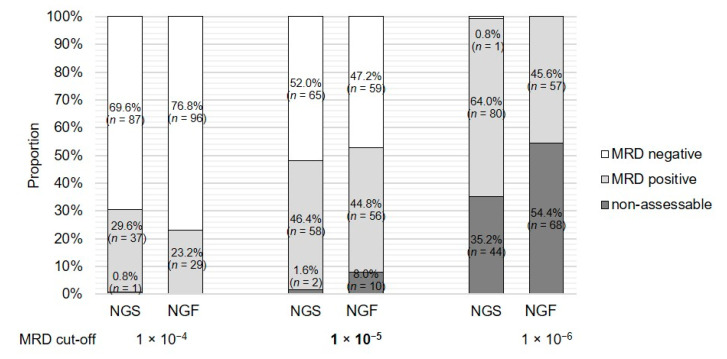
MRD rates based on different MRD cut-offs and key metrics from NGS and MFC analyses. The figure illustrates the changes in absolute numbers of nonassessable, MRD-positive and MRD-negative results depending on the choice of MRD cut-off for the overall cohort. The decision is attributed to the limit of detection (LOD). Concordances and discordances between NGS and MFC are not considered.

The effect of assigning different MRD cut-offs can impressively be demonstrated by the assignment of the MRD status (positive vs. negative vs. nonassessable) based on the sample TL and LOD at the consensus MRD cut-offs (Figure 2). Stepwise decreasing of the MRD cut-off from 1 × 10^−4^ to 1 × 10^−6^ resulted in a decrease in MRD-negative cases from 69.6% (*n* = 87) to 0.8% *(n* = 1) in the NGS analysis and from 76.8% (*n* = 96) to 0.0% (*n* = 0) in the MFC analysis. However, the number of nonassessable cases due to missed LOD increased by 34.4% in the NGS analysis (from 0.8%, *n* = 1 to 35.2%, *n* = 44) and by 54.4% (from 0.0%, *n* = 0 to 54.4%, *n* = 68) in the MFC analysis. Based on the median LOD and percentage of nonassessable cases, 1 × 10^−5^ was chosen as the best-fit MRD cut-off for further analysis in the current study (median LOD 1.7 × 10^−6^ in NGS and 6.0 × 10^−6^ in MFC; nonassessable cases 1.6%, *n* = 2 in NGS and 8.0%, *n* = 10 in MFC).

### 2.2. The Concordance of NGS and MFC MRD Results Reaches Almost 70%

**Figure 3:**
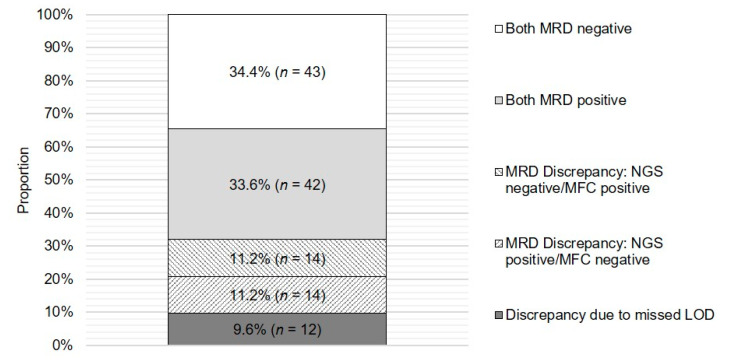
Proportion of concordant and discordant MRD results between NGS and MFC analyses. Relative and absolute numbers of concordant and discordant MRD results obtained from NGS and MFC assays at an MRD cut-off of 1 × 10^−5^ are shown. LOD, limit of detection.

At an MRD cut-off of 1 × 10^−5^, concordant MRD-positive results were found in 33.6% (*n* = 42) of cases, and concordant MRD-negative results were found in 34.4% (*n* = 43), resulting in an overall concordance proportion of 68.0% (*n* = 85). Discordant results—NGS MRD positivity/MFC MRD negativity and vice versa—were found in 11.2% (*n* = 14) of patients in each direction, resulting in an overall discordance rate of 22.4% (*n* = 28). Discordancy due to undercut LOD by either of the two methods was estimated to be 9.6% (*n* = 12; Figure 3). Excluding discordant results caused by undercut LOD by either of the methods, Cohen’s kappa coefficient (κ) for interrater agreement between the MRD status of the two methods was 0.536 (*n* = 113, *p* < 0.001).

### 2.3. MRD Status Obtained with Both Assays Corresponds to Serological Response

To assess whether the obtained MRD results are plausible, we calculated the proportion of MRD-negative and positive cases at the MRD cut-off of 1 × 10^−5^, with regard to serological remission status, for each assay separately. MRD results obtained with both techniques adequately corresponded to the serological response.

Serological remission status at the time of MRD assessment was available in 125 patients. Fifty-four, 31, 24, 7 and 1 patients reached a complete response (CR), near complete response (nCR), very good partial response (VGPR), partial response (PR) and minimal response (MR), respectively (Table 1).

**Figure 4:**
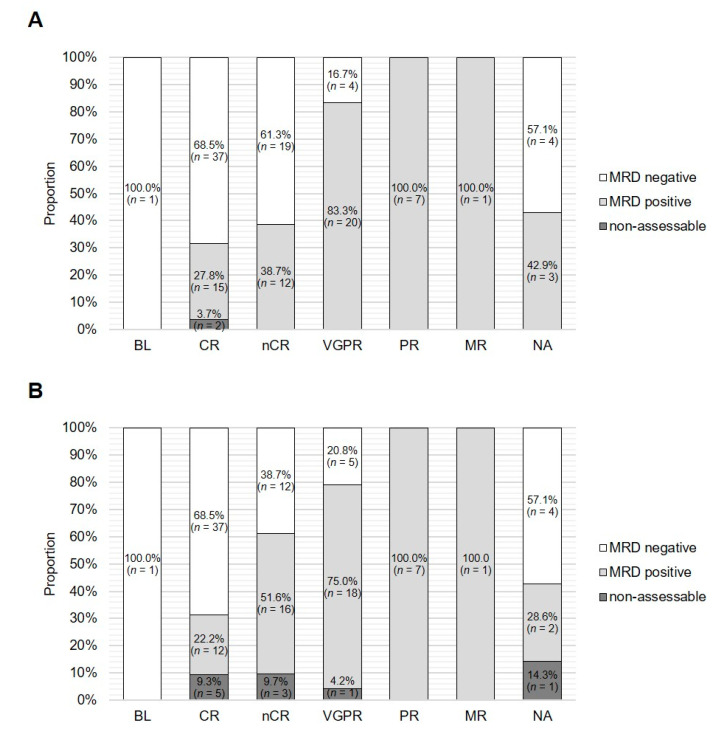
MRD status assessed by NGS and MFC according to serological response. The proportion of MRD-positive, negative and nonassessable cases at the MRD cut-off of 1 × 10^−5^ is shown according to the serological remission status for (**A**) NGS and (**B**) NGF. BL, baseline; CR, complete remission; NA, not available; nCR, near complete remission; PR, partial remission; VGPR, very good partial remission.

The proportion of NGS MRD-negative cases decreased from 68.5% (*n* = 37) to 61.3% (*n* = 19) and to 16.7% (*n* = 4) at serological CR, nCR and VGPR, respectively, and no NGS MRD-negative cases were found in the case of serological PR and MR. Overall, 55.1% (*n* = 60/109) of patients with a serological response ≥VGPR showed BM MRD negativity by NGS (Figure 4A).

Similarly, when assessed by MFC, the proportion of MRD-negative cases decreased from 68.5% (*n* = 37) to 38.7% (*n* = 12) and to 20.8% (*n* = 5) at serological CR, nCR and VGPR, and no MRD-negative cases were found at serological PR or MR. Overall, 49.5% (*n* = 54/109) of patients with a serological response ≥VGPR showed BM MRD negativity by MFC (Figure 4B).

### 2.4. The Tumor Load of MRD-Positive Cases Is Comparable with Both Assays

**Figure 5:**
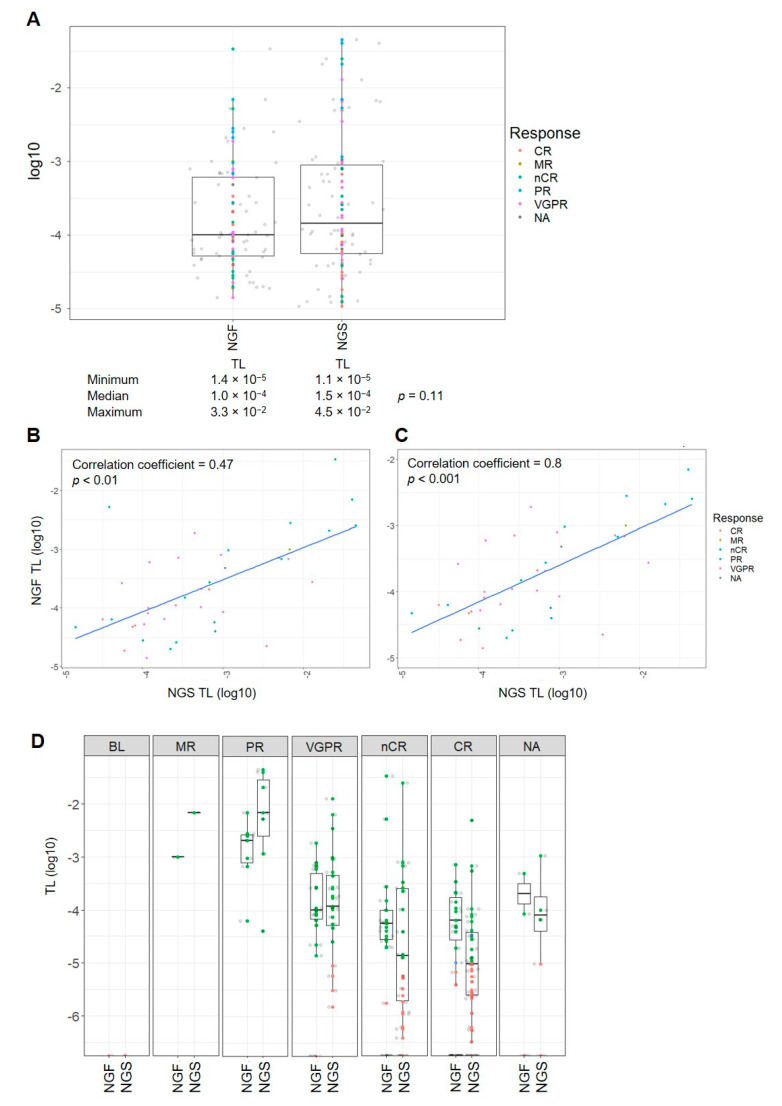
Tumor load in MRD-positive cases. The figure shows the tumor loads (TLs) of NGS/NGF MRD-positive cases at an MRD cut-off of 1 × 10^−5^. The results are presented (**A**) cumulatively for all these cases and (**D**) according to serological response; all comparison *p*-values > 0.05; green, MRD-positive; red, MRD-negative; blue, MRD-nonassessable due to a missed LOD. The correlation between the TLs of the MRD-positive pairs assessed by NGS and MFC is shown for (**B**) all pairs and (**C**) after removal of two extreme outliers. BL, baseline; CR, complete remission; MR, minimal response; NA, not available; nCR, near complete remission; PR, partial remission; TL, tumor load; VGPR, very good partial remission.

Both methods were compared regarding the quantification of aberrant cells/cell equivalents in NGS/MFC MRD-positive cases (*n* = 58 and 56, respectively) at the MRD cut-off of 1 × 10^−5^. The median TL in these cases was 1.5 × 10^−4^ (1.1 × 10^−5^–4.5 × 10^−2^) with NGS and 1.0 × 10^−4^ (1.4 × 10^−5^–3.3 × 10^−2^) with MFC. No statistically significant difference was found between the TLs assessed by NGS and MFC (*p* = 0.11, Figure 5A). Pearson’s product-moment correlation of the TLs of the 42 concordant MRD-positive pairs resulted in a correlation coefficient of 0.47 between NGS and MFC (*n* = 42, *p* < 0.01, Figure 5B). Removing two extreme outliers from the MRD-positive pairs, the estimate for the correlation coefficient was corrected to 0.8, indicating a good correlation (*n* = 40, *p* < 0.001, Figure 5C). Additionally, among the difference response categories, no significant difference between the TLs assessed by NGS and MFC was found (*p* > 0.05, respectively, Figure 5D).

## 3. Discussion

Herein, we provide a detailed evaluation of MRD results obtained in 125 MM patients with two different laboratory methods. The aim of this study was to compare the two methods—Adaptive Biotechnologies NGS MRD assay and Cytognos MFC MM MRD—in terms of concordance and suggest an evaluation algorithm for a step-by-step interpretation of MM MRD results. Our study contributes to the analysis and interpretation of data, closing the gap between technical and prognostic reports on MM MRD. Technical differences concerning applicability, processing requirements, sample quality, turnaround time, cost and others have been discussed and described in detail by Mina et al. (2020) [13] among others.

The MRD result as clinical response parameter of an individual patient is only affected by its own LOD and LOQ and is assessable without an MRD cut-off. In clinical trial settings, the collective LOD and LOQ levels strongly affect the number of assessable MRD analyses of a study cohort at different MRD levels. Shifting the study-wide MRD cut-off towards lower values not only influences the decision regarding MRD positivity or negativity but might also increase the number of nonassessable cases due to undercutting the LOD and LOQ.

In our study, undercutting the LOD with an MRD cut-off of 1 × 10^−5^ resulted in a nonassessable MRD status that affected less than 10% of all cases (1.6%, *n* = 2 in NGS and 8.0%, *n* = 10 in MFC) and accounted for 10% of the discrepancy between paired samples. Despite the low number of cases with nonassessable MRD status, this result pointed to an important issue of MRD analysis. While consensus recommendations on MM MRD analysis and reporting reasonably requests MRD levels, LOD and LOQ in order to give precise context and achieve interstudy comparability of the published results [3,12,14,15], these guidelines fail to emphasize the role and consequences of the definition of MRD cut-offs. While each sample has an individual LOD and LOQ—as a function of the individual number of cells tested—which may be translated into an individual MRD status, global MRD cut-offs affect the analyzability of the whole study cohort. This became obvious as we had to define the optimal data cut-off for the definition of MRD-positive and MRD-negative status in the current study. Our data showed that shifting the MRD cut-off from 1 × 10^−5^ towards a higher sensitivity of 1 × 10^−6^ resulted in a greater proportion of nonassessable cases due to an undercut LOD (35.2%, *n* = 44, median LOD 1.7 × 10^−6^ in NGS and 54.4%, *n* = 68, median LOD 6.0 × 10^−6^ in MFC), leaving the study short by 50% of cases if 1 × 10^−6^ was chosen as the study-wide MRD cut-off. Accordingly, 1 × 10^−5^ was chosen as the best-fit MRD cut-off for evaluation as it met the international guidelines and resulted in a tolerable proportion of nonassessable cases in both methods (1.6%, *n* = 2 in NGS and 8.0%, *n* = 10 in MFC).

Overall, our study provides important perspectives on previous publications reporting that both NGS and NGF-based MRD assays can reach a sensitivity of up to one tumor cell per 1,000,000 BM cells (10^−6^) [1,3]. Specifically, our results highlight the fact that these claims are based on optimal sample quantity, quality and sample processing, which may or may not be applicable in the clinical setting. Our study did not evaluate the general applicability of the methods or potential effects of pre-analytic sample handling, such as BM collection, time from sampling until analysis and shipping conditions [13,16,17].

However, in line with other authors, we would like to emphasize the importance of sample amount and quality, which should be carefully considered when planning a trial or applying MRD diagnostics in clinical routine since they ultimately and significantly affect each sample’s individual LOD and LOQ values and the sensitivity that can be reached, respectively [13,16,17,18]. Particularly in the context of (multicenter) clinical trials, the aspect of undercut study-specific or international MRD cut-off values, e.g., <1 × 10^−5^ as defined by the European Medicines Agency (EMA) [12], might consequently lead to non-analyzability of the trial as a result of an excessive number of nonassessable cases.

The impact of sample quality is especially high for MFC/NGF since viable cells and not DNA are required for analysis. This renders the technique challenging for logistics in multicenter studies. For sequencing-based methods, samples—either in full or in the form of DNA extracts—can be stored and shipped for analysis more easily. Furthermore, the number of cells required to reach comparable sensitivities is higher for MFC/NGF, as we also demonstrated in our study (median 1.1 × 10^6^ cell equivalents to reach a median LOD of 1.7 × 10^−6^ in NGS, median 5.0 × 10^6^ cells to reach a median LOD of 6.0 × 10^−6^ in NGF). For clinical decision-making, it might be of importance to be able to confirm a negative result as a true negative, which is difficult if the sample material is not suitable for storage.

Recently, attention has been drawn to hemodilution effects in BM aspirates, leading to an overestimation of analyzed cells as a “background” for tumor cell components and false sensitivity estimates. While in IG sequencing-based methods, hemodilution cannot be assessed, the latest MFC/NGF assays indicate hemodilution by a decreased percentage of mast cells in the sample [2,14]. The issue of how to avoid hemodilution in the first place and still gather enough cells for sensitive analysis at the same time has been discussed but is yet unresolved.

In the current analysis, we identified a decreasing proportion of MRD-positive cases with deeper serological response with both methods. Through NGS, 43.1% (47/109) of patients with ≥VGPR were MRD-positive. This is in line with the 43% (17/40 cases) MRD positivity rate reported by Korde et al. in patients with ≥VGPR [19]. Through MFC, 42.2% (46/109) of patients with ≥VGPR were MRD-positive. The proportion of MRD-positive patients achieving ≥VGPR by MFC reported by Flores-Montero et al. was 47% (52/110 patients) and therefore very similar to the results obtained in the current analysis [2]. We could not identify any statistically significant differences in the TL between NGS and MFC in MRD-positive cases assessed by both methods, neither for the whole cohort nor according to the serological remission status. For concordant MRD-positive cases (*n* = 42), the correlation coefficient of 0.47 revealed a moderate TL correlation between both methods, and after removing two extreme outliers, the correlation coefficient was corrected to 0.8, indicating good correlation. This result was within the range of the correlation estimate obtained by Flores-Montero et al., who reported a good correlation (0.62) between the percentage of residual aberrant plasma cells by NGS and MFC in a cohort of 27 patients [2]. In light of sample availability, turnaround time, cost and foremost the lower number of cells necessary for high sensitivity in NGS, it might be of use to implement a step-wise diagnostics algorithm for clinical response analysis which might take the following form: i) if serological response VGPR or better is reached, MRD diagnostics by MCF/NGF should be performed; ii) if MFC/NGF result negative at 10^−5^, MRD diagnostics by NGS should be performed. To prove such an algorithm successful and applicable in clinical routine, prospective clinical trials are necessary. Such trials could further implement less invasive sample types such as peripheral blood for pre-screening before BM MRD analysis and/or imaging technologies for monitoring.

## 4. Materials and Methods

### 4.1. Patient Cohort and Sample Matching

MRD samples from MM patients who were randomized to participate in the multicenter prospective phase III HD6 trial on the “Effect of Elotuzumab in Velcade, Revlimid, and Dexamethasone (VRD) Induction/Consolidation and Lenalidomide Maintenance” (16 March 2015; NCT02495922, EudraCT: 2014-003079-40) conducted by the German-Speaking Myeloma Multicenter Group (GMMG) were evaluated [20,21]. All patients provided written informed consent before participating in the study. Approval was obtained by the ethics committee of the University of Heidelberg in collaboration with the participating centers’ ethics committees (16 June 2015; AFmu-562/2014). The baseline clinical characteristics of the included MM patients are summarized in Table 1.

Aiming to compare the NGS and MFC assays and, at this time, not taking into consideration the evaluation treatment efficiency of the trial population, 125 patients were selected for, whom MRD was measured with both MRD detection methods at the first timepoint of assessment until March 2018. Importantly, in the GMMG-HD6, sample collection for MRD evaluation was triggered by serological response criteria and not according to a fixed timepoint. The remission status was assessed according to the International Myeloma Working Group (IMWG) response criteria [22]. Since patients reach complete remission according to serological response at different treatment phases, the timepoints of MRD assessment in the analyzed patient cohort vary from post-induction to post-transplant and post-consolidation therapy, as indicated in Table 1.

MRD samples (heparin anticoagulant, 15 mL first pull preferable) were obtained by BM aspiration from os ilium (spina iliaca posterior superior, no image guidance) and were shipped from participating centers to the Laboratory for Hemato-Oncological Diagnostics and the Molecular Biology Laboratory (Heidelberg University Hospital, Heidelberg, Germany) for further analysis. One MRD sample was collected per patient for NGS and MFC assessment to ensure a similar sample quality for both MFC and NGS analysis.

### 4.2. MRD Assessment by NGS

DNA was extracted after density gradient separation of mononuclear cells from fresh BM aspirate, which was then stored at −20 °C until NGS analysis. NGS analysis was performed at Adaptive Biotechnologies (Seattle, WA, USA) in accordance with previous reports [3,19,23,24]. For initial clone identification, 625 ng genomic DNA per patient was sent for analysis. For MRD determination, 9 µg genomic DNA per patient was sent. An analysis report was provided by Adaptive Biotechnologies.

### 4.3. MRD Assessment by MFC

**Figure 6:**
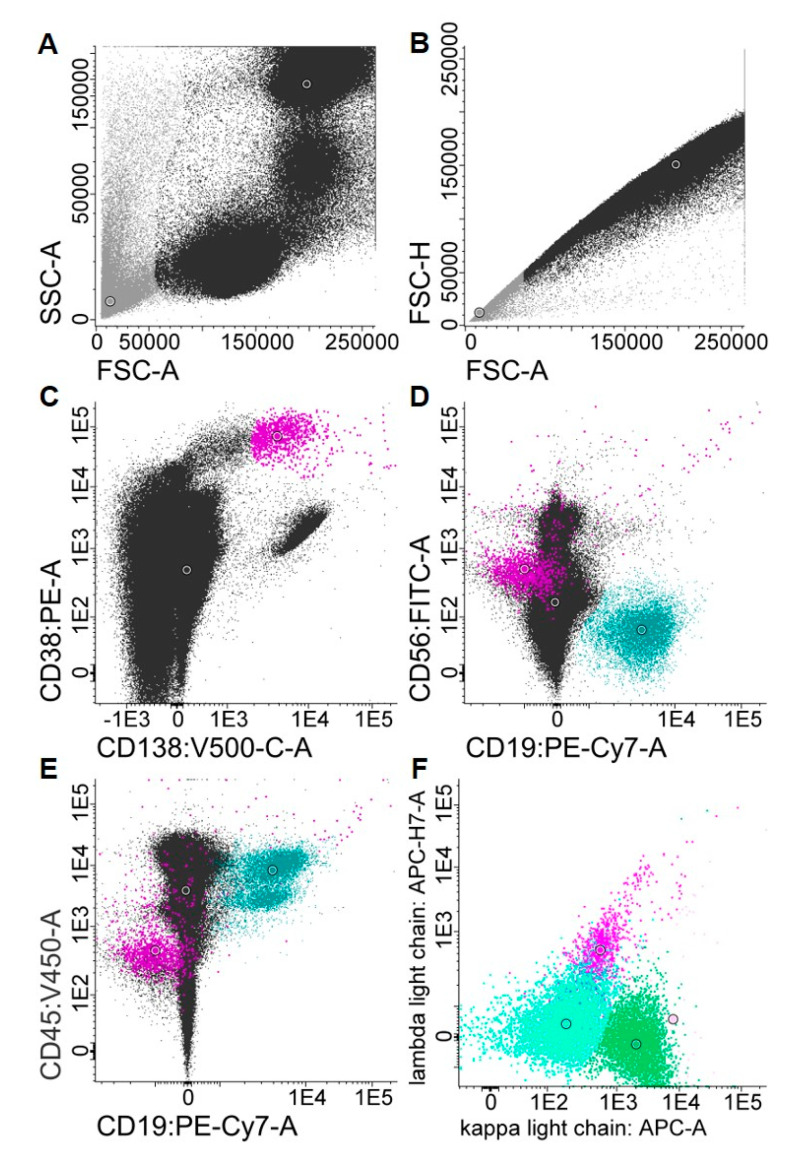
Gating strategy for the identification of aberrant plasma cells. After exclusion of debris (gray, **A**) and duplets (gray, **B**), plasma cells are defined as the CD38- and CD138-positive population (purple, **C**) among the leukocytes (black). Aberrant plasma cells are usually CD56-positive, CD19-low and CD45-low (**D**,**E**). Moreover, aberrant plasma cells show light chain restriction (in this case, lambda, **F**). Non-aberrant plasma cells do not display a light chain restriction, similar to B-cells (green) that display a regular distribution with regard to the kappa and lambda light chains (**F**). Gating was performed with Infinicyt Flow Cytometry Software. SSC-A, side scatter area; FSC-A, forward scatter area; FSC-H, forward scatter height.

Flow cytometry was performed according to the highly standardized MFC approach developed and described by the Spanish Myeloma Collaborative Group using a commercially available EuroFlow 8-color 2-tube MM MRD Kit (Cytognos, Salamanca, Spain) [2]. Tube one contained multiepitope CD38-FITC, CD56-PE (clone C5.9), CD45-PerCP-Cyanine5.5 (clone EO1), CD19-PE-Cyanine7 (clone 19-1), CD117-APC (clone 104D2) and CD81-APC-C750 (clone M38) antibodies. Tube two contained multiepitope CD38-FITC, CD56-PE (clone C5.9), CD45-PerCP-Cyanine5.5 (clone EO1), CD19-PE-Cyanine7 (clone 19-1), cytoplasmic polyclonal immunoglobulin (Ig) κ-APC goat and cytoplasmic polyclonal Igλ-APC-C750 antibodies. Drop-in CD27 Brilliant Violet 510 (clone O323, Biolegend, San Diego, USA) and CD138 Brilliant Violet 421 (clone MI15, BD, Heidelberg, Germany) antibodies were added to tubes one and two. Measurements were performed on a cell analyzer (BD, Heidelberg, Germany) after implementation of the EuroFlow Standard Operating Protocol for Instrument Setup and Compensation in FACSDiva (BD Biosciences, San Jose, CA, USA). Depending on sample quality, we aimed to acquire 10^7^ events for optimal MRD sensitivity metrics. Final data analysis was performed in Infinicyt 2.0 (Cytognos, Salamanca, Spain). An automated gating and identification tool (Cytognos, Salamanca, Spain) was used to support the identification of MM cells. Plasma cells were identified based on the coexpression of CD38 and CD138 antigens. An aberrant plasma cell expression profile was defined as CD45-low/negative, CD56-positive, CD19-negative and light chain-restricted (Figure 6) [25,26].

### 4.4. Calculation of MRD Key Metrics

TL, LOD, usually called the sensitivity level, a prerequisite for the “MRD-positive” vs. “MRD-negative” vs. “nonassessable” decision, and LOQ, the basis for the numerical quantification of the tumor load, were calculated as sample-specific MRD key metrics individually for the NGS and MFC analyses.

For the NGS assay, LOD was defined as the sample MRD frequency for which the probability of falsely claiming the absence of MRD is 5% and experimentally determined to be 1.9/number of tested cells. LOQ was defined as the lowest NGS MRD sample MRD frequency that can be quantitatively determined with an accuracy of 70% total error and was determined to be 2.39. The analysis report provided by Adaptive Biotechnologies was translated to match the commonly used negative exponential notation (e.g., one-in-one million, i.e., 1 × 10^−6^). Adaptive Biotechnologies provided LOD, LOQ and TL values scaled per million. For the report, the calculation of NGS LOD is determined by considering the uniqueness of the rearrangement, the predefined LOD correction value of 1.9 and normalized to per million cells assayed.

NGS LOQ was calculated considering the uniqueness of the rearrangement, the predefined value of 2.4 and normalized to per million cells assayed, and NGS TL was calculated as sequence count/number of tested cells.

In MFC, the LOD and LOQ were calculated according to the “Consensus Guidelines on Plasma Cell Myeloma Minimal Residual Disease Analysis and Reporting”: MFC LOD = 30/total number of events acquired, and MFC LOQ = 50/total number of events acquired. MFC TL was calculated as the number of aberrant plasma cells/total number of nucleated cells acquired.

**Figure 7:**
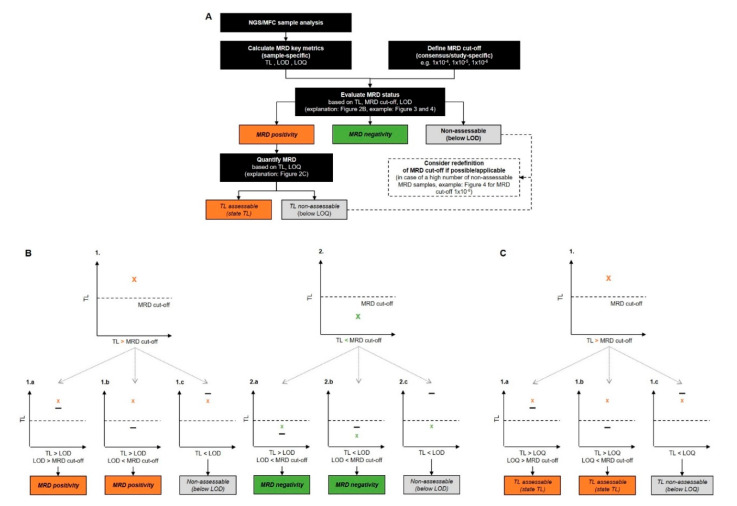
Data evaluation workflow for NGS and NGF MM MRD results. The general data evaluation workflow is shown in (**A**), while (**B**) and (**C**) specify the decision regarding MRD positivity/negativity and the final statement of the TL. (**A**) Upon NGS/MFC sample analysis, sample-specific MRD key metrics (TL, LOD, LOQ) are calculated according to the MRD method, as described in the Methods section of the manuscript. Based on the calculated sample-specific metrics and a specific MRD cut-off (consensus and/or study-specific), the MRD status is evaluated in the first step. If the evaluation of the MRD status reveals MRD positivity, the TL is assessed. These two steps might reveal a high number of nonassessable cases because of a missed LOD or LOQ due to too few analyzed cells/cell equivalents. Therefore, a redefining of the study-specific MRD cut-off might be necessary/considered. (**B**) A sample with a TL above the MRD cut-off (B.1.) might be generally considered MRD-positive. However, this is only true for samples with a TL above the LOD (B.1.a and B.1.b). If the TL is lower than the LOD, the case should be referred to as “nonassessable” (B.1.c). A sample with a TL below the MRD cut-off (B.2) might be generally considered MRD-negative. However, this is only true for samples with an LOD below the MRD cut-off (B.2.a and B.2.b). If the LOD is higher than the MRD cut-off, the case should be referred to as “nonassessable” regarding the MRD status (B.2.c). (**C**) The TL is determined when the first step of the MRD evaluation reveals MRD positivity. In an MRD-positive sample, the TL can be generally stated. However, this is only true for samples with a TL above the LOQ (C.1.a and C.1.b). If the TL is below the LOQ, the case should be referred to as “nonassessable” regarding TL quantification. In this case, the sample is MRD-positive, but the TL cannot be stated. X tumor load (TL); ▬ sample limit of detection (LOD) or quantification (LOQ); --- predefined minimal residual disease (MRD) cut-off.

To perform a detailed comparison of the NGS and MFC results, different MRD cut-offs (1 × 10^−4^, 2 × 10^−5^, 1 × 10^−5^ and 1 × 10^−6^) were considered according to previous reports and institutional specifications (European Medicines Agency, EMA) [12,14,18]. The detailed data evaluation workflow is presented in Figure 7.

### 4.5. Statistical Analysis

Statistical analyses were performed in the statistical software environment R/R-Studio (The R Foundation for Statistical Computing Platform) using the ggplot2, plyr and irr packages [27,28,29]. For descriptive statistics, categorical data such as MRD status are presented in absolute numbers and percentages. Continuous data such as the MRD metrics TL, LOD and LOQ are presented as medians and ranges in exponential notation and plotted on a log_10_ transformed scale. Cohen’s kappa coefficient (κ) for two raters was calculated as an interrater agreement measure for the MRD statuses assessed by NGS and MFC. The TLs between NGS and MFC were compared by a two-sided paired t-test. Correlations between the TLs obtained with NGS and MFC were estimated by Pearson’s product-moment correlation. A *p*-value ≤ 0.05 was considered statistically significant.

## 5. Conclusions

In conclusion, our study demonstrated good concordance between MRD status and tumor load between NGS and MFC at a threshold of 10^−5^. However, the number of total analyzed events and therefore the resulting LOD and LOQ might significantly influence the choice of the best-fit MRD cut-off for study with a tolerable proportion of nonassessable cases.

## Figures and Tables

**Table 1:** Patient characteristics at first diagnosis, timepoints of MRD assessment and serological remission.

Number of Patients, *n*	125
Median age, years (range)	59 (33–70)
Gender, *n* (%)	
Female	53 (42.4)
Male	72 (57.6)
Heavy chain type, *n* (%)	
IgA	24 (19.2)
IgD	1 (0.8)
IgE	1 (0.8)
IgG	67 (53.6)
IgM	2 (1.6)
LC	30 (24.0)
Light chain type, *n* (%)	
κ	78 (62.4)
λ	47 (37.6)
Median BM plasma cells, % (range)	
Cytology	40 (2–100)
Histopathology	40 (1–98)
Median β2-microglobulin, mg/L (range)	3.9 (1.1–24)
Median Albumin, g/L (range)	36.9 (20.3–49.3)
ISS, *n* (%)	
I	44 (35.2)
II	53 (42.4)
III	28 (22.4)
Timepoint of MRD assessment, *n* (%)	
Baseline	1 (0.8)
Pos Iinduction	22 (17.6)
Post Mobilization	1 (0.8)
Post-HD-CT/ASCT 1	57 (45.6)
Post-HD-CT/ASCT 2	2 (1.6)
Post Consolidation	41 (32.8)
NA	1 (0.8)
Serological remission, *n* (%)	
Baseline	1 (0.8)
CR	54 (43.2)
nCR	31 (24.8)
VGPR	24 (19.2)
PR	7 (5.6)
MR	1 (0.8)
NA	7 (5.6)

ASCT, autologous stem cell transplantation; BM, bone marrow; CR, complete response; HD-CT, high-dose chemotherapy; ISS, International Staging System, nCR, near complete response; MR, minimal response; PR, partial response; VGPR, very good partial response; NA, not available.
